# Induction of HCA587-Specific Antitumor Immunity with HCA587 Protein Formulated with CpG and ISCOM in Mice

**DOI:** 10.1371/journal.pone.0047219

**Published:** 2012-10-11

**Authors:** Juanjuan Chen, Lijie Zhang, Weigang Wen, Jiaqing Hao, Pumei Zeng, Xiaoping Qian, Yu Zhang, Yanhui Yin

**Affiliations:** Department of Immunology, School of Basic Medical Sciences, and Key Laboratory of Medical Immunology of Ministry of Health, Peking University Health Science Center, Beijing, People’s Republic of China; Federal University of São Paulo, Brazil

## Abstract

HCA587 (also known as MAGE-C2) is a “cancer-testis” antigen highly expressed in a number of malignancies with unique immunological properties, making it a promising target for tumor immunotherapy. In this report, we demonstrated that HCA587 protein, when formulated with adjuvants CpG–containing oligodeoxynucleotides (CpG ODN) and ISCOM, was capable of inducing a potent cellular and humoral immune response as indicated by the presence of a large number of HCA587-specific, IFN-γ-producing CD4^+^ T cells and high levels of HCA587-specific antibodies. More importantly, vaccination with HCA587 conferred protection against challenge with HCA587-expressing B16 melanoma in prophylactic and therapeutic settings. In analysis of the mechanisms underlying the protective effect, we showed that the vaccination was followed by enhanced accumulation of tumor-infiltrating lymphocytes (TILs) with enrichment of conventional CD4^+^ T cells but reduced representation of Treg cells. Further, the antitumor effect was largely abrogated in mice either depleted of CD4^+^ T cells or deficient for IFN-γ. These results indicate that HCA587 protein vaccine possesses evident antitumor activity in a mouse model and holds promise for treatment of human cancers.

## Introduction

Over the last two decades, antigen-specific immunotherapy has emerged as an alternative to systemic chemotherapies with the advantage of less toxicity and durable clinical responses. The recent approval by the US FDA of sipuleucel-T consisting of PA2024 fusion protein-loaded APCs for prostate cancer therapy [1], [2] represents a major breakthrough for cancer vaccine-based therapy. It opens the door for the clinical application of cancer vaccines in the treatment of advanced cancers. However, main challenges in developing effective therapeutic vaccines are the selection of appropriate target antigens and effective adjuvants to stimulate a robust and durable immune response [3], [4].

Cancer-testis (CT) antigens are characterized by a unique pattern of tissue expression, highly expressed in cancer cells but absent from normal adult tissues other than immunoprivileged germ-line tissues [5]. This tissue restriction makes them ideal targets for cancer immunotherapy. Up to now, a total of 110 CT gene families have been reported and entered into the CT database established recently by the Ludwig Institute for Cancer Research (http://www.cta.lncc.br) [6]. However, only a few CT gene encoding proteins have been proved to be immunogenic so far, of which, MAGE-A3 and NY-ESO-1 are being actively tested in multiple clinical trials for their therapeutic potentials using different combinations of adjuvants, immunomodulators, and vaccine delivery method [7], [8], [9].

As a novel CT antigen, HCA587 (also known as MAGE-C2) has been proved to be one of the most immunogenic tumor antigens [10], [11], [12]. To date, nine cytotoxic T lymphocyte (CTL) and four help T cell (Th) epitopes derived from HCA587 have been identified [13], [14], [15], [16], [17], [18]. HCA587 is abnormally expressed in a wide variety of malignancies, including hepatocellular carcinoma, melanoma, bladder cancer, breast cancer, sarcoma and lung cancer, etc [19], [20], [21]. Immunohistochemical studies revealed that HCA587 protein (34–37%) was more frequently detected than NY-ESO-1 (7.3–23.3%) and MAGE-A3 (7%) in hepatocellular carcinoma patients [19], [22], [23], [24], [25]. Intriguingly, in the melanoma patients who showed tumor regression after MAGE-A3 vaccination, CTLs against MAGE-C2 were highly present in the blood, with a frequency of 10–10,000 times higher than that of anti-MAGE-A3 CTLs. Moreover, anti-MAGE-C2 CTLs showed a much higher enrichment in metastases, 10,000 times more frequent than anti-vaccine T cells. Therefore, T cell clones recognizing MAGE-C2 antigen were considered to play an important role in tumor regression in the MAGE-A3 vaccinated melanoma patients [8], [11], [26]. These findings make HCA587 a particular attractive target in cancer immunotherapy.

In order to explore the therapeutic potential of HCA587, the present study was focused on the development of vaccination strategies and the evaluation of the antitumor activities of HCA587-based cancer vaccines in a mouse model. We demonstrated that HCA587 protein, in combination with adjuvants CpG ODN and ISCOM, elicited strong humoral and cell-mediated immune responses. More importantly, the immunized animals showed resistance to the challenge of HCA587-expressing tumors. This antitumor activity was dependent on CD4^+^ but not CD8^+^ T cells, and IFN-γ appeared to be indispensable for the protection. This represents the first exploitation of HCA587-based immune-interventions in mice.

**Figure 1:**
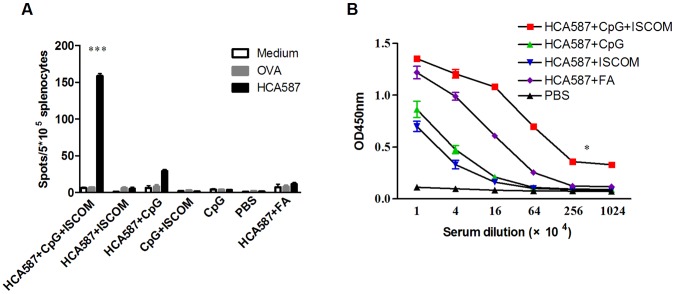
Immunization with HCA587 protein induces antigen specific cellular and humoral immune responses. C57BL/6 mice (nine per group) were immunized s.c. with 100 µl of HCA587 protein (10 µg) in combination with various adjuvants including CFA (50 µl), CpG ODN (12 µg), ISCOM (12 µg), or CpG ODN plus ISCOM, and boosted 21 days later. For the CFA group, IFA was used for the second immunization. Splenocytes and sera were harvested 14 days after the boost. (A) Number of IFN-γ-producing splenocytes. Splenocytes were restimulated with HCA587 protein for 20 h. IFN-γ-producing cells were detected by ELISPOT assay. Data are presented as mean ± SD. The irrelevant protein ovalbumin (OVA) and medium alone served as controls. ***, *P*<0.0001, compared with other groups. (B) Serum antibodies against HCA587 protein. Levels of HCA587-specific antibodies were measured by ELISA. *, *P*<0.05, HCA587+ CpG + ISCOM in comparison with HCA587+ CpG or HCA587+ ISCOM.

## Materials and Methods

### Ethics Statement

All animal studies were carried out in strict accordance with the recommendations in the Guide for the Care and Use of Laboratory Animals of the National Institutes of Health. The protocol was approved by the Animal Care and Use Committees of Peking University Health Science Center (Permit Number: IRB00001052-0711). All efforts were made to minimize suffering. When the animal suffering was too great, humane endpoints were applied.

**Figure 2:**
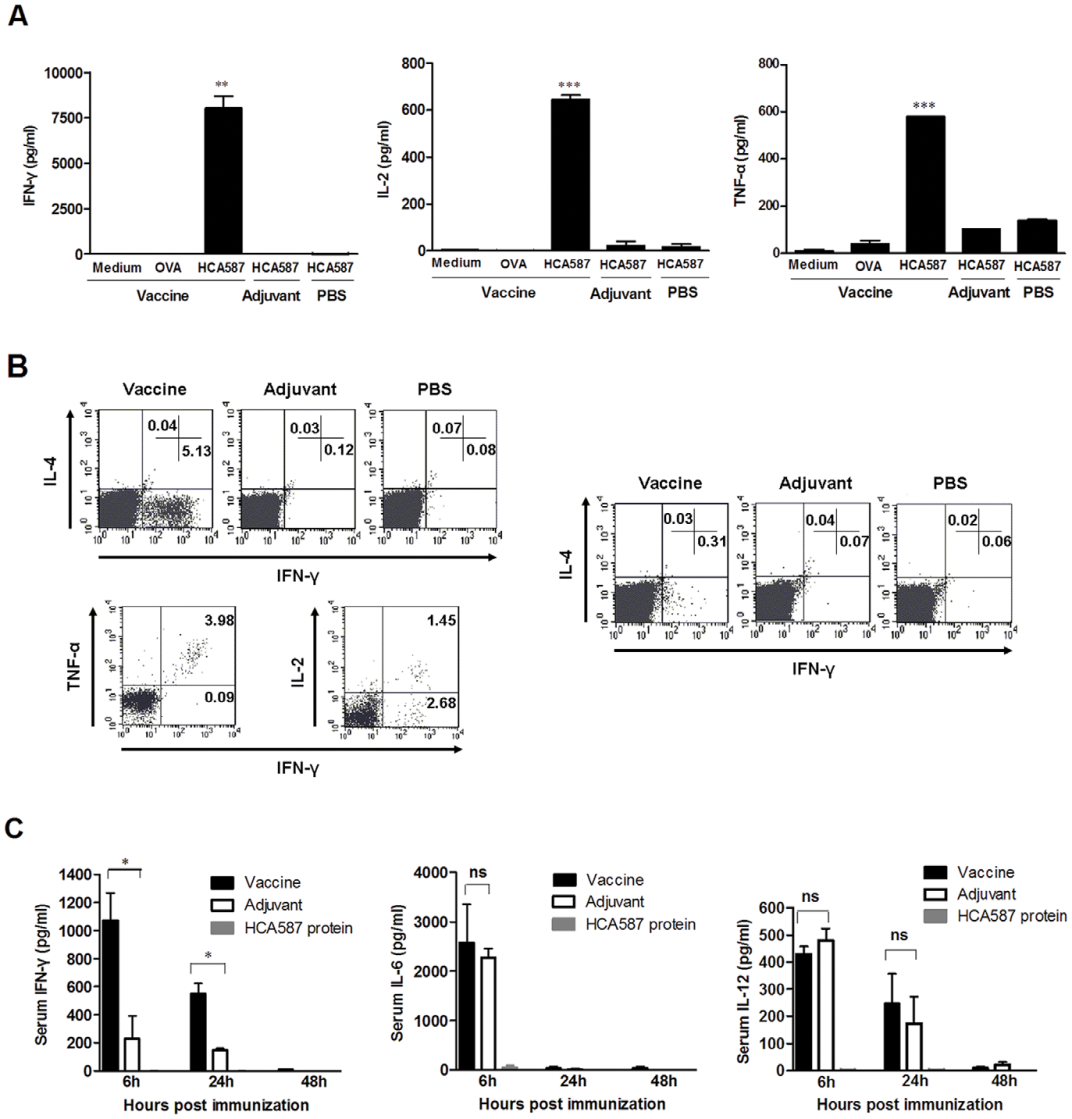
HCA587 protein vaccination triggers Th1-type responses. Mice (nine per group) were immunized with HCA587+ CpG + ISCOM, CpG + ISCOM, or PBS, and boosted 21 days later. (A) Splenocytes were harvested 14 days after the boost and cultured with medium alone, ovalbumin (OVA) or HCA587 protein for 24 h. Cytokine levels in the supernatants were determined using commercial ELISA kits for IFN-γ, IL-2, and TNF-α. ***, *P*<0.001; **, *P*<0.01, compared with other groups. (B) Splenocytes were stimulated with HCA587 protein for 24 h. Intracellular staining was performed for IFN-γ, IL-4, TNF-α and/or IL-2 following surface staining for CD4 and CD8. The experiments were performed at least 3 times with similar results. A representative profile of cytokines produced by CD4^+^ (*Left*) and CD8^+^ (*Right*) T cells is shown. (C) Serum cytokine levels. Sera were collected at 6, 24 and 48 h after the boost and analyzed for levels of IFN-γ, IL-6,and IL-12 using ELISA kits. *Columns*, mean values; *bars*, SE. *, *P*<0.05; ns, *P* > 0.05.

### Mice

C57BL/6 mice were obtained from the Laboratory Animal Center of Peking University. IFN-γ knockout mice on a C57BL/6 background were purchased from Model Animal Research Center of Nanjing University. Mice were housed in microisolators under standard pathogen-free conditions. Mice were between 6 and 8 weeks of age at the start of the experiments.

### Antibodies

The anti-HCA587 monoclonal antibody (mAb), clone LX-CT10.5 [27], was provided by Professor Boquan Jin (Fourth Military Medical University, Xi’an, China). The anti-CD4 mAb GK1.5 and anti-CD8 mAb 53-6.7 were affinity purified using protein G-Sepharose columns (GE Healthcare, Piscataway, NJ). Fluorescence labeled antibodies specific for CD4 and CD8 were purchased from BD Biosciences (San Jose, CA), antibodies specific for IFN-γ, IL-2, IL-4, TNF-α, and NK1.1 were obtained from Biolegend (San Diego, CA). In all experiments, control mAbs of appropriate isotypes were included.

**Figure 3:**
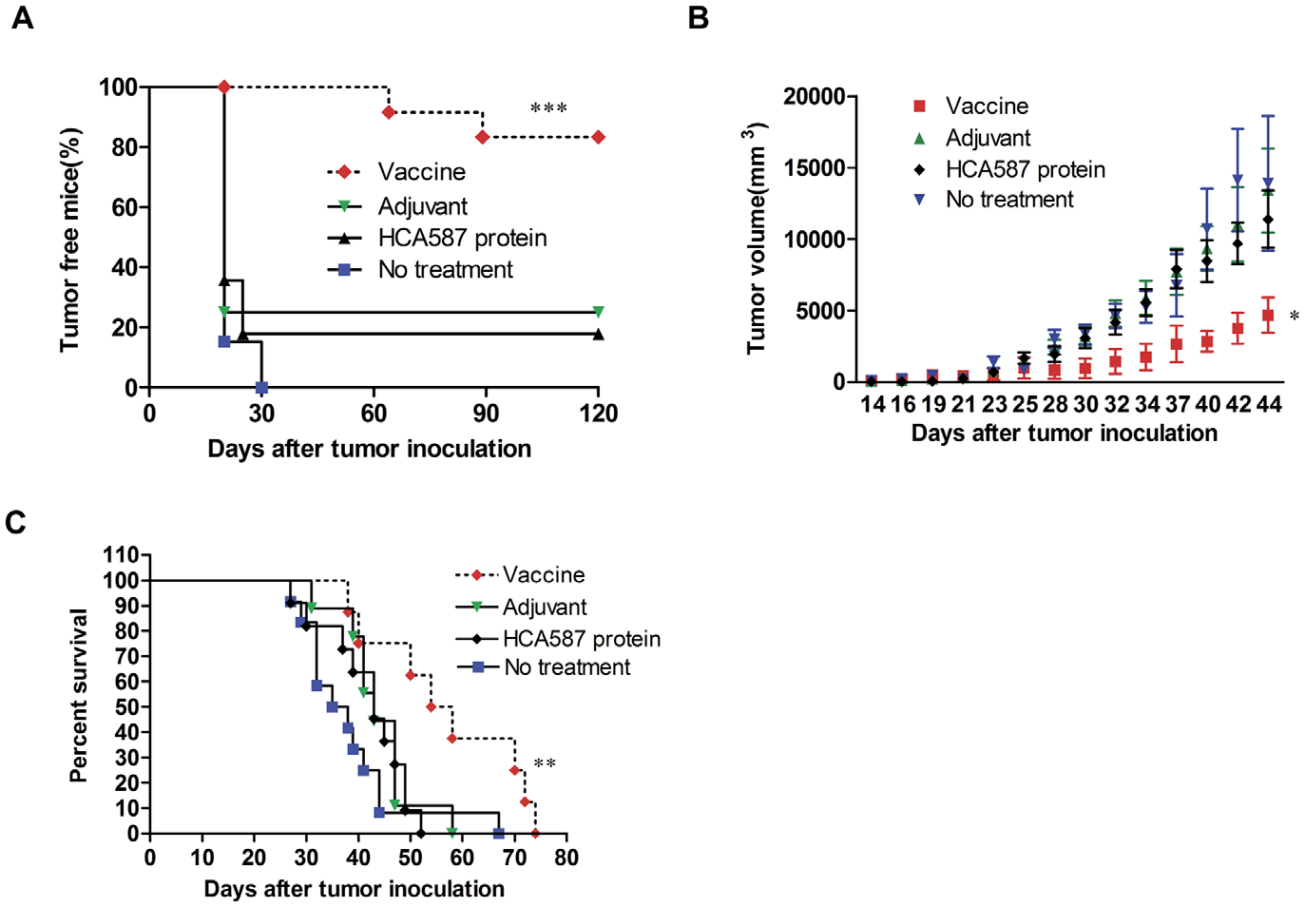
Antitumor efficacy of HCA587-based vaccine in murine melanoma model. (A) *In vivo* tumor protection experiments. C57BL/6 mice (twelve per group) were immunized with HCA587 protein vaccine, or CpG + ISCOM, or protein alone, twice at a 3-week interval. Two weeks after the final immunization, mice were challenged with 1×10^4^ B16-HCA587 tumor cells. Tumor growth was monitored every 2 to 3 days with an endpoint at day 120. ***, *P*<0.0001, compared with other groups. (B, C) *In vivo* tumor treatment experiments. C57BL/6 mice were inoculated with 1×10^4^ B16-HCA587 tumor cells, and left untreated or treated with HCA587 protein vaccine, HCA587 protein alone, or adjuvants on day 7 and 28 post inoculation. Tumor sizes were measured every 2–3 days. (B) Tumor size (n = 8–12 per group). *Points*, mean of tumor volume; *bars*, SE. *, *P*<0.05, compared with other groups. (C) Survival curve of tumor-bearing mice (n = 8–12 per group). **, *P*<0.01, compared with other groups.

### Cell Lines

B16 melanoma cell line, a gift from Dr. Jingrong Cui (Peking University, Beijing, China), was originally obtained from Cell Bank of Shanghai Institute of Cell Biology, Chinese Academy of Sciences, Shanghai, China. The B16 melanoma cell was transfected with plasmid pEGFP-C1 containing the full-length HCA587 cDNA sequence, using Lipofectamine 2000 (Invitrogen, Cralsbad, CA), as recommended by the manufacturer. Stable transfectants were selected in G418 (1.3 mg/ml) and cloned by limiting dilution. HCA587 expression of individual clones was confirmed by Western blot analysis. The tumor cells were maintained in DMEM with 10% heat-inactivated fetal bovine serum (Invitrogen). On the day of tumor challenge, tumor cells were harvested by trypsinization, washed extensively, and then resuspended in PBS to the designated density for injection. Cell viability was assessed by trypan blue exclusion.

**Figure 4:**
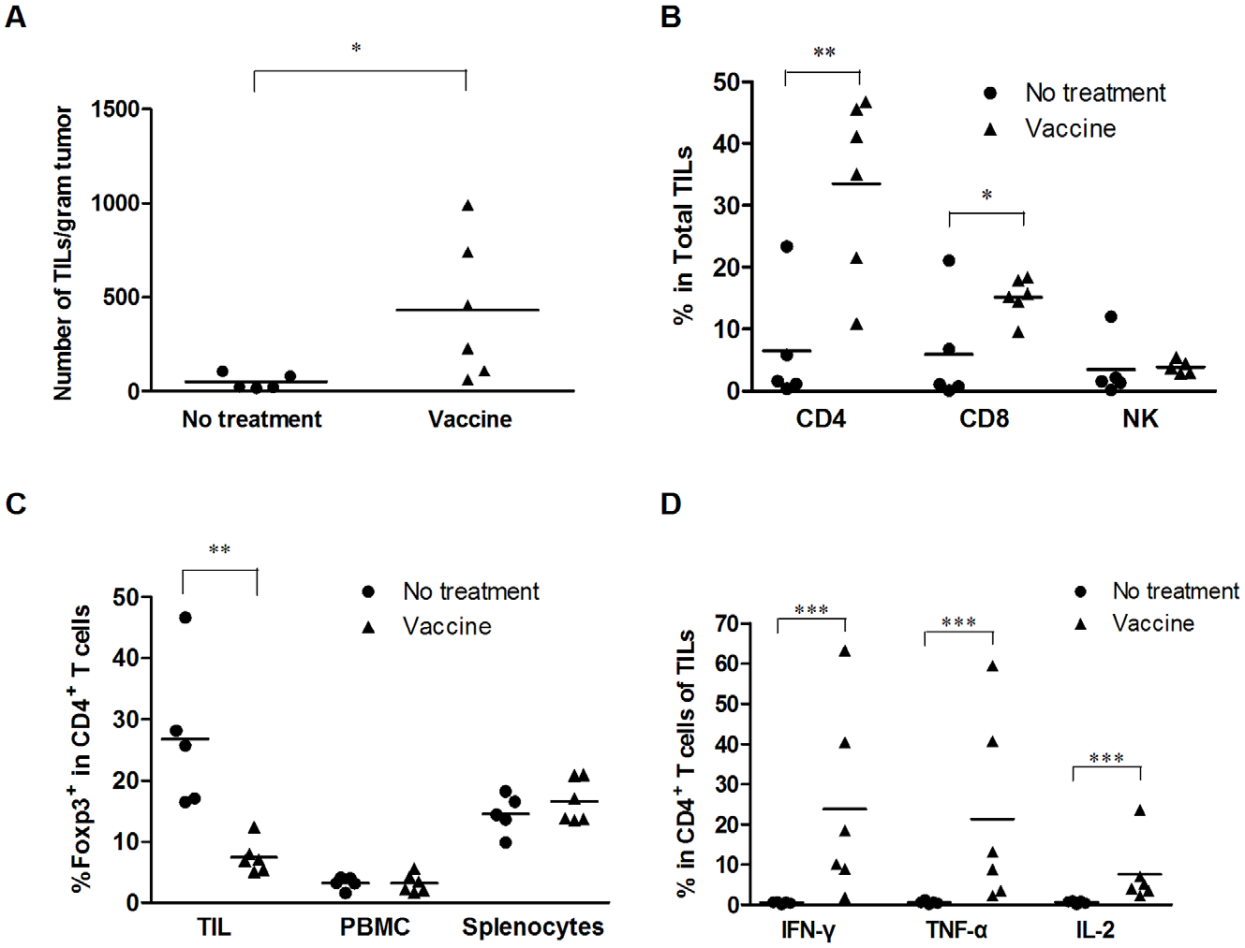
HCA587 protein vaccine enhances intratumoral accumulation of HCA587-responsive CD4^+^ T lymphocytes. C57BL/6 mice (5–6 per group) were inoculated with B16-HCA587 cells, and left untreated or treated with HCA587 protein vaccine on day 7 and 28 post inoculation. Two weeks after the last treatment, tumor tissues were collected and TILs were isolated. (A) Number of TILs. Data are presented as the cell number per gram of tumor mass. (B) Representation of major subsets of immune cells in TILs. TILs were analyzed by flow cytometry and the percentage of CD4^+^ T, CD8^+^ T and NK1.1^+^ cells were calculated. (C) Percentage of Foxp3^+^ cells in the CD4^+^ populations in the tumor, PBMCs, or spleen. (D) HCA587-responsive CD4^+^ T cells in TILs. TILs were plated at a density of 5×10^6^ cells per well in 24-well dishes in RPMI1640 with 10% FBS and 10µg/ml HCA587 protein at 37°C and 5% CO2 for 24 hours, and 10 µg/ml brefeldin A was added 18 hours before harvesting the cells from the culture. Intracellular staining was then performed to detect the production of IFN-γ, TNF-α and IL-2. The percentages of cytokine-producing cells are presented among intratumoral CD4^+^ T cells. Each dot or triangle represents an individual sample, and horizontal bars denote the mean. *, *P*<0.05; **, *P*<0.01, ***, *P*<0.001, compared with untreated group.

### Antigen and Adjuvants

Recombinant human HCA587 protein was prepared by Crown Bioscience, Inc (Beijing, China). Briefly, the expressing vector pGEX-6p-1-HCA587 was constructed and expressed in the *E. coli* strain BL21 (DE3). The fusion protein was first affinity-captured using Glutathione Sepharose 4B (GE Healthcare). After removal of the GST-tag by cleavage with PPase, the recombinant HCA587 protein was further purified by ion-exchange chromatography (HiTrap Q HP, GE Healthcare) and gel filtration (Superdex 200, GE Healthcare). The purity of recombinant HCA587 protein were analyzed using reverse phase-high performance liquid chromatography (RP-HPLC) and the full sequence of recombinant HCA587 protein was verified by tandem MS. The purity of HCA587 protein used for vaccine was more than 95%, with endotoxin at a level below 3.1 units/100 µg protein. All-phosphorothioate modified CpG oligonucleotide (CpG ODN) 1826 (5′ –TCCATGACGTTCCTGACGTT-3′) was synthesized by the Shanghai Sangon Biological Engineering & Technology and Service (Shanghai, China). Immune-stimulating complex (ISCOM) AbISCO 100 was purchased from Corporation Isconova AB, Sweden. Complete Freund’s Adjuvant (CFA) and Incomplete Freund’s Adjuvant (IFA) were purchased from Sigma-Aldrich (St. Louis, MO).

**Figure 5:**
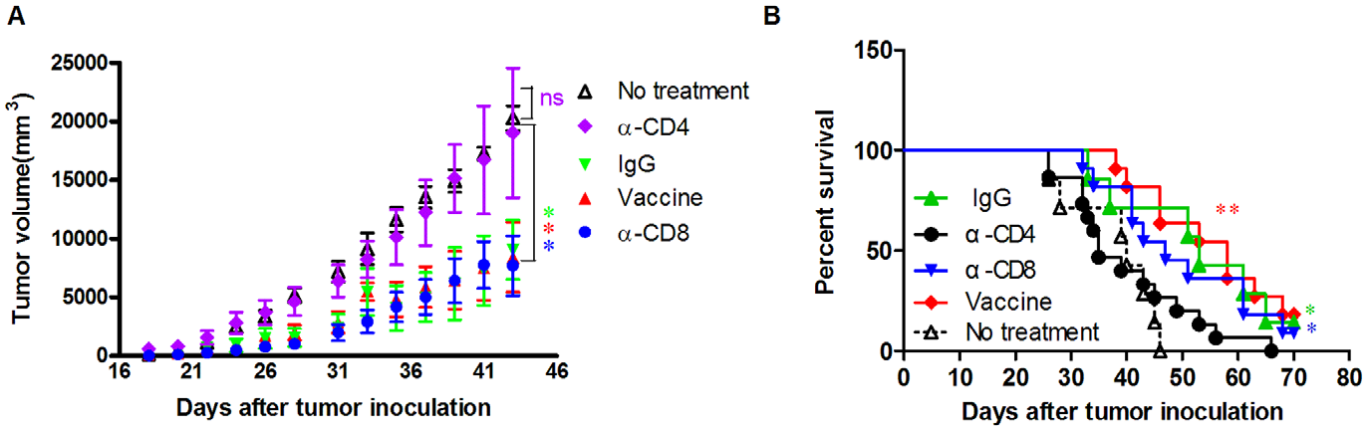
The antitumor effect of HCA587 protein vaccine is mediated by CD4^+^ T lymphocytes. Tumor inoculation and vaccination was performed as described above except that some groups of the mice (10–15 for each group) received additional injections of 500 µg of GK1.5 (rat anti-mouse CD4 mAb), 53-6.7 (rat anti-mouse CD8 mAb), or isotype controls 1 day before the vaccination and twice a week thereafter for a total of eight times. The group of mice (n = 7) received tumor cells but no vaccination served as controls. (A) Tumor size. *Points*, mean of tumor volume; *bars*, SE. *, *P*<0.05; ns, *P* > 0.05, compared with untreated control group. (B) Survival curve of the mice. **, *P*<0.01; *, *P*<0.05, compared with untreated control group.

### Immunization

Mice were immunized s.c. at the base of the tail with 10 µg of HCA587 protein. Different adjuvants or combinations of them were tested for their ability to induce optimal cellular and/or humoral responses, including CFA (50 µl), CpG ODN (12 µg), ISCOM (12 µg), or CpG ODN plus ISCOM. A total volume of 100 µl was administrated using PBS as a vehicle. Each animal received two injections at a 3 week interval. For the CFA group, IFA was used for the second immunization. Controls were set up by immunizing age-matched mice with PBS, HCA587 protein alone, or adjuvants alone.

**Figure 6:**
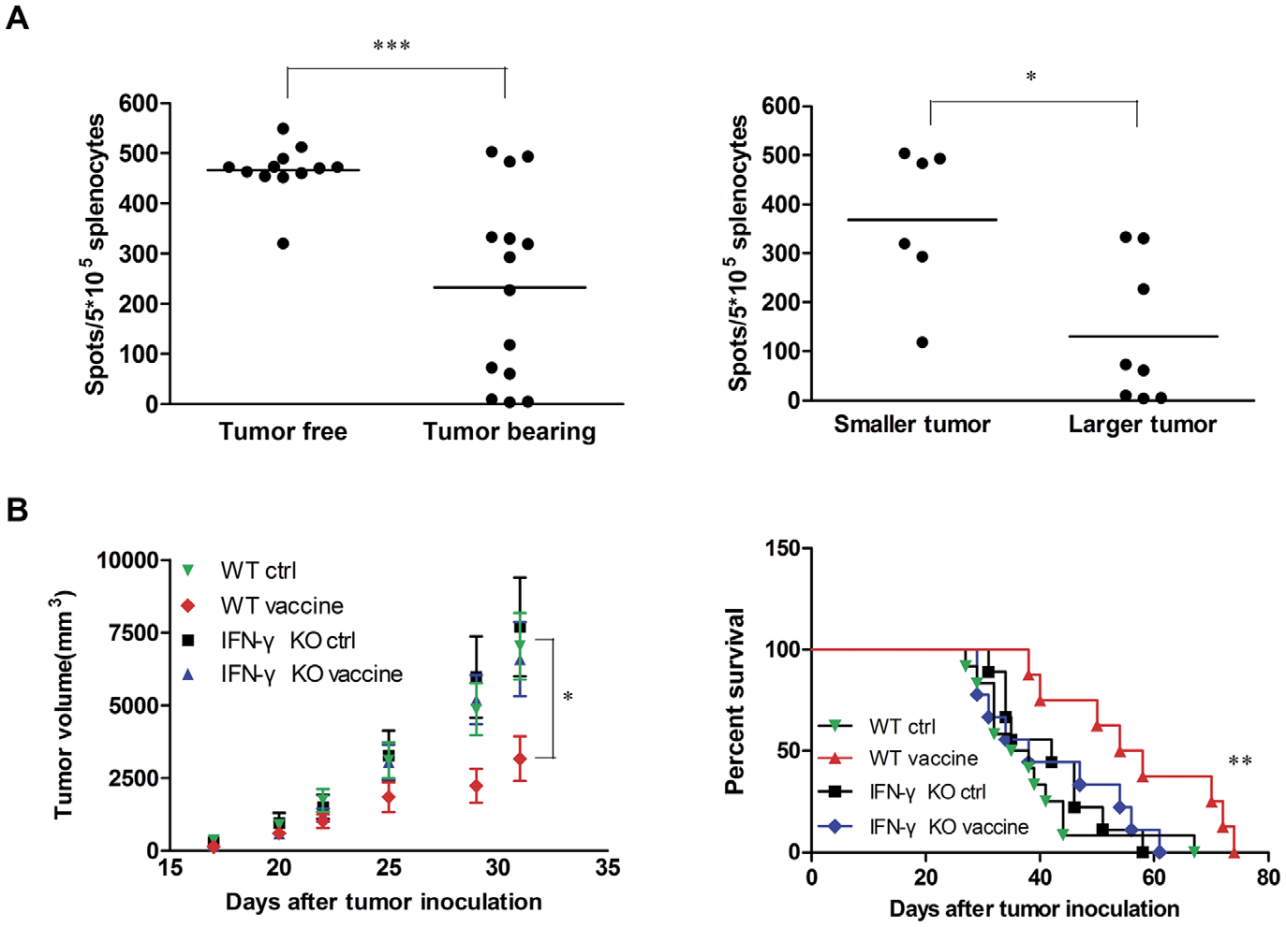
IFN-γ is required for the antitumor effect of HCA587 protein vaccine. (A) Correlation of the frequency of IFN-γ-producing cells and tumor development. Tumor inoculation and vaccination was performed as described above. Splenocytes were isolated 2 weeks after the last boost, and analyzed for IFN-γ production in response to HCA587 restimulation by ELISPOT assay. The *left* panel shows the frequency of IFN-γ-producing splenocytes in tumor-free (n = 12) versus tumor-bearing mice (n = 14). The tumor-bearing mice were further divided into two groups: small tumor with a volume <7000 mm^3^ (n = 6) and large tumor with a volume >7000 mm^3^ (n = 8), and the frequency of IFN-γ-producing splenocytes in these two groups is shown in the *right* panel. (B) Abrogation of vaccine-induced protection in IFN-γ KO mice. IFN-γ KO mice on C57BL/6 background and C57BL/6 (WT) mice (8–12 mice for each group) inoculated with B16-HCA587 cells were treated with HCA587 protein vaccine or left untreated. Tumor growth and mouse survival was closely monitored. The tumor volume is presented on the *left* as mean ± SE, and the survival curve is shown on the *right*. *** *P*<0.001; ** *P*<0.01; *, *P*<0.05.

### Antibody ELISA Assay

Serum samples were collected from the mice at different time points. HCA587-specific antibody titers were detected using enzyme-linked immunosorbent assay (ELISA). Briefly, ELISA plates were coated with HCA587 protein at 1 µg/ml in PBS, and then blocked with 2% bovine serum albumin. Serially diluted mouse serum samples were added into each well and incubated at 37°C for 2 hours. Mouse IgG bound on the plates was detected by horseradish peroxidase-conjugated goat anti-mouse IgG (Promega, Madison, WI) using tetramethylbenzidine (TMB; Tiangen Biotechnology Corporation, Beijing, China) as a peroxidase substrate. The reaction was stopped by the addition of 2 M H_2_SO_4_, and the absorbance was read at 450 nm. To detect mouse serum IgG subclass, biotin-labeled anti-IgG1 and anti-IgG2c antibodies (Bethyl, Montgomery, TX) and streptavidin-conjugated HRP (BD, San Jose, CA) were used.

### IFN-γ ELISPOT Assay

After lysing RBCs, splenocytes were resuspended at a density of 5×10^6^/ml, and 100 µl of this suspension was then incubated with HCA587 protein (2.5 µg/ml) or ovalbumin (OVA; 2.5 µg/ml, Sigma-aldrich) in ELISPOT plates coated with anti-IFN-γ capture Abs (Mabtech, Nacka, Sweden). After incubation for 20 hours at 37°C, cells were removed, and the plates were developed with a biotinylated anti-mouse IFN-γ detecting Abs and streptavidin-alkaline phosphatase (Mabtech). The dark violet spots displayed on the plate membranes were automatically counted with the ELISPOT reader (Sage Creation, Beijing, China).

### Cytokine Assay

RBC-depleted splenocytes (5×10^6^/ml) from vaccinated mice were prepared and stimulated with HCA587 protein (10 µg/ml) or ovalbumin (10 µg/ml) for 24 hours at 37°C. The supernatants were collected and analyzed for IL-4, IL-5, IFN-γ, IL-2, TNF-α, IL-10, and IL-17 with commercial available ELISA kits (Dakewe Biotechnology, Beijing, China) according to the manufacture’s procedures. Similar procedures were followed to measure the levels of IFN-γ, IL-12, IL-6, IL-10, and TNF-α in the serum of immunized mice.

For intracellular cytokine staining, brefeldin A (10 µg/ml; Biolegend) was added 18 hours before harvesting the cells from the culture. The cells were first surface stained for CD4 and CD8, then fixed and permeabilized (Biolegend) according to the manufacturer’s instruction, followed by staining with fluorochrome-labeled antibodies against IFN-γ, IL-4, IL-2, TNF-α, or isotype-matched control antibodies. Data were collected using a FACSCalibur cytometer (BD, San Jose, CA) and analyzed using CellQuest Pro software.

### Evaluation of the Antitumor Activity of HCA587 Protein Vaccine *in Vivo*

The protective effect of vaccination was assessed using two different models. In the prophylactic model, mice first received two injections of the vaccine at a three-week interval. Two weeks after the last vaccination, mice were challenged with 1×10^4^ B16-HCA587 tumor cells by s.c. injection on the flank. In the therapeutic model, mice were first inoculated with tumor cells, and the vaccine was administrated 1 and 4 weeks after the inoculation. Mouse survival was monitored daily, and tumor volume was measured every 2 to 3 days with a caliper, and calculated using the following formula: long axis× (short axis) ^2^× 0.52.

### Phenotypic and Functional Analysis of Tumor-infiltrating Lymphocytes (TILs)

Tumor-bearing mice were sacrificed around 6 weeks after tumor cell inoculation, and TILs were isolated as previously described [28]. Number of TILs per gram of tumor mass was calculated. Following surface staining with anti-NK1.1-PE, anti-CD4-PerCP-Cy5.5, and anti-CD8-APC, intracellular staining of Foxp3 was performed using a Foxp3 kit (eBioscience, San Diego, CA) according to the manufacturer’s instructions. Cytokines produced by CD4^+^ TILs were analyzed by intracellular cytokine staining as described above after ex vivo stimulation with HCA587 protein for 24 h.

### *In vivo* Cell Depletion

To delete CD4^+^ or CD8^+^ cell, mice received i.p. injections of 500 µg of GK1.5 (rat anti-mouse CD4 mAb), 53-6.7 (rat anti-mouse CD8 mAb), or isotype controls 1day before the first administration of vaccines and twice a week thereafter for 4 weeks. The efficacy of cell depletion was confirmed by flow cytometric analysis of spleen.

### Statistical Analysis

All analysis and graphics were done using GraphPad Prism, version 4 for PC (GraphPad Software, San Diego, CA). Experiments were repeated two to three times with multiple samples for each time. The statistical significance of differential findings was determined using Student’s *t* test. Differences in the survival of mice were analyzed using the Kaplan-Meier method, and groups were compared using the log-rank test. Statistical significance was based on a value of *P*<0.05.

## Results

### HCA587 Protein Formulated with CpG ODN and ISCOM Stimulates Strong Humoral and Cell-mediated Responses

The intensity and the type of immune responses to the immunizing antigen are heavily dependent upon the co-administration of adjuvants. Freund’s adjuvant (FA) tends to induce good antibody production but poor cellular response [29]. CpG ODN and ISCOM, on the other hand, display the capacity to enhance both humoral and cellular responses, and are therefore included as integrated components in a number of vaccines under development [30], [31]. To identify the appropriate adjuvants for the HCA587-based cancer vaccine, we immunized C57BL/6 mice with the recombinant HCA587 protein formulated with different adjuvants or their combinations. Fourteen days after the second immunization, splenocytes were prepared and analyzed for the presence of HCA587-specific, IFN-γ-secreting cells using an ELISPOT assay. As depicted in Figure 1A, a much enhanced cellular response was observed upon co-application of CpG ODN and ISCOM with HCA587 protein, with a 6-fold increase in IFN-γ-secreting cells over HCA587+ CpG ODN vaccination group. We also tested the generation of HCA587 specific antibodies under different conditions. As expected, FA was a potent inducer of antibody response. Although CPG ODN or ISCOM evoked a relatively poor response when applied alone, their co-administration generated the highest anti-HCA587 antibody titer (Figure 1B). Furthermore, we analyzed the durability of the immune response. Significant anti-HCA587 B-cell and T-cell responses were observed even 6 months after the final immunization (Figure S1), indicating that the immunity is long lived.

Together, these data indicate that HCA587 protein formulated with CpG ODN and ISCOM (hereinafter referred to as HCA587 protein vaccine) is capable of inducing potent and long lasting cellular and humoral immune responses.

### Vaccination with HCA587 Protein Vaccine Preferentially Induces a Th1-type Response

To explore the predominant type of cellular response induced by the HCA587 protein vaccine, we examined the profile of cytokines secreted by splenocytes of immunized mice after *in vitro* stimulation with HCA587. High levels of Th1-type cytokines, including IFN-γ, IL-2 and TNF-α were present in the culture supernatant of splenocytes prepared from vaccinated mice and rechallenged with HCA587 protein (Figure 2A). In contrast, IL-4, IL-5, IL-10 and IL-17, which are characteristic of Th2- and Th17-type cells, respectively, were not detected in any of the cultures except that IL-4 was detected in very low concentration in a few cases (Figure S2).

Intracellular cytokine staining further revealed that the IFN-γ-producing cells were mainly present in the CD4^+^ fraction, accounting for approximately 5% of all CD4^+^ T cells in the culture (Figure 2B). A small but significant percentage of IFN-γ-producing cells were also detected among CD8^+^ T cells (Figure 2B). The NK cell fraction, on the other hand contained no IFN-γ-producing cell (data not shown). Of note, the majority of IFN-γ-producing CD4^+^ T cells were also positive for TNF-α, while only one third of them produced IL-2 as well (Figure 2B). It is worth mentioning that we failed to detect any IL-4-producing cell in either CD4^+^ or CD8^+^ populations (Figure 2B), and IL-10-producing CD4^+^ T cells were also not detectable (Figure S3).

The serum cytokine levels were also monitored in the immunized mice. Significant levels of IFN-γ was only detected in mice receiving the fully formulated HCA587 vaccine, which peaked at 6 hour after the second immunization, gradually decreased thereafter, and returned to the baseline at 48 hour (Figure 2C). To the contrary, elevated levels of IL-6 and IL-12 were seen in mice treated either with the protein vaccine or the adjuvant (CpG ODN plus ISCOM) (Figure 2C), but there were no significant differences between the two groups, indicating that the elevation is largely attributable to the adjuvant effect. Again, no significant secretion of IL-10 was seen in any of the groups (data not shown).

In addition, the mice immunized with HCA587 protein vaccine generated a HCA587-specific IgG2c isotype response which was significantly higher than the HCA587-specific IgG1 isotype (Figure S4).

Overall, these results suggest that the HCA587 protein vaccine induces a Th1-biased response.

### HCA587 Vaccination Confers Antitumor Activity in Mouse Models

Tumor protection experiments were next performed to determine if the observed HCA587-specific immune responses might be translated into antitumor effects *in vivo*. The mouse B16 melanoma model was used in the present study as melanoma is one of the human tumors known to have high levels of HCA587 expression [20]. The B16 cell line, which does not express the human HCA587, was transfected with the full-length HCA587 gene. A clone showing stable expression of HCA587 (B16-HCA587) (Figure S5) was selected for subsequent studies. In the prophylactic model, C57BL/6 mice were vaccinated on day 0 and day 21, and inoculated with B16-HCA587 melanoma cells on day 35. The HCA587 protein vaccine-treated mice showed a significant decrease in tumor incidence, with 83% (10/12) of mice remaining tumor-free at the end of the experiment (120 days after tumor inoculation), which was in comparison to 17% (2/12) in the protein-only group and 25% (3/12) in the adjuvant group (Figure 3A).

The HCA587 protein vaccine was further tested for its efficacy in a therapeutic model. Mice were inoculated with 1×10^4^ B16-HCA587 cells, and then divided into four groups, each containing 12 mice. One group was left untreated, and the other three were treated at day 7 and day 28 either with HCA587 protein alone, CpG ODN plus ISCOM, or the fully formulated vaccine. Subsequent analysis of tumor volume and survival was performed with tumor-bearing mice in each group. As shown in Figure 3B, the tumor growth was retarded in the vaccine group compared with all other groups. In addition, vaccination led to significantly prolonged survival of the tumor-bearing mice (Figure 3C). In contrast to the profound impact on HCA587-expressing B16 cells, treatment with the HCA587 protein vaccine showed no effect on the growth of B16 cells expressing the green fluorescent protein (GFP) (Figure S6), suggesting that the antitumor effect is highly antigen-specific.

Taken together, these data demonstrate that immunity induced by the HCA587 protein vaccine has potent antigen-specific antitumor activity *in vivo*.

### HCA587 Protein Vaccine Enhances Accumulation and Activation of Tumor-infiltrating Lymphocytes (TILs)

To characterize the antitumor response induced by HCA587 protein vaccine, we performed phenotypic and functional analyses of intratumoral lymphocytes in tumor-bearing mice. Compared with the untreated group, lymphocyte infiltration within the tumor, as measured by cell number per gram of tumor, was found to be significantly enhanced in the vaccine group (Figure 4A). In addition to the increase of total cellularity, the composition of tumor-infiltrating lymphocytes (TILs) was drastically altered. Relative to less than 10% in untreated tumors, CD4^+^ T cells accounted for more than 30% of infiltrating lymphocytes in vaccine-treated tumors (Figure 4B). The percentage of CD8^+^ T cells was also seen to be rising in the vaccine group, whereas NK cells were similarly represented in the two groups (Figure 4B). In contrast to the general increase of CD4^+^ T cells, the frequency of Foxp3 expressing cells within the CD4^+^ population was 4-fold lower in the vaccine-treated tumor than in the untreated tumor, while it was comparable in peripheral blood and spleen between the two groups (Figure 4C).

It was next determined whether the lymphocytes present in tumor tissues were functional. To this end, freshly isolated intratumoral lymphocytes were assessed, using intracellular cytokine staining, for their capacity to produce cytokines upon stimulation with the HCA587 protein. As expected, no HCA587-responsive cell was detected in untreated tumors. On the other hand, significant percentages of CD4^+^ T cells from vaccine-treated tumors were capable of producing IFN-γ, TNF-α, and IL-2 in response to HCA587 stimulation (Figure 4D). No cytokine-producing cells, however, were present in CD8^+^ T or NK cells (data not shown).

These results clearly demonstrate that the vaccine treatment greatly enhances intratumoral accumulation of CD4^+^ T cells, many of which are ready to respond to the immunizing antigen. In addition, vaccination leads to reduced presence of Treg cells.

### The Vaccine-induced Antitumor Activity Is CD4^+^ T Cell- and IFN-γ-dependent

To determine the cell population critical for the antitumor activity induced by the HCA587 protein vaccine, we performed *in vivo* depletion of CD8^+^ or CD4^+^ cells using specific antibodies. The depletion of CD4^+^ T cells abrogated the antitumor effect, while CD8^+^ T cells depletion showed no influence on the efficacy of the HCA587 protein vaccine (Figure 5 A and B).

There is accumulating evidence that IFN-γ plays a vital role in antitumor immunity [32], [33]. We also noticed an inverse correlation between the number of IFN-γ-producing cells and tumor formation and progression in HCA587 vaccine-treated mice. The tumor-free mice showed high percentage of IFN-γ-producing splenocytes compared with the tumor-bearing mice, and the mice with smaller tumors tended to have more such cells than the mice with larger tumor volume (Figure 6A). Similar correlation was also observed between IFN-γ-producing tumor-infiltrating CD4^+^ T cells and tumor growth (data not shown).

To clarify the role of IFN-γ in the antitumor activity conferred by the HCA587 protein vaccine, IFN-γ knockout mice were engaged. While the antitumor effect was readily observable in wild type mice, tumor growth inhibition and survival prolongation did not occur in B16-HCA587-inoculated IFN-γ knockout mice after treatment with the vaccine (Figure 6B), suggesting that IFN-γ is required for the vaccine-induced effect.

## Discussion

HCA587, cloned by SEREX from the hepatocellular carcinoma-derived cDNA libraries by our group [21], is widely expressed in various types of human tumors but not in normal adult tissues apart from testis. The strong immunogenicity and restricted expression of HCA587 protein make it an ideal candidate for specific cancer immunity [10]–[20]. A murine homologue of HCA587, known as MAGE-K, is exclusively expressed in mouse testis and cross-reacts with anti-HCA587 antibodies (Figure S7). In the present study, we explored the potential of HCA587 as a target for cancer immunotherapy in murine tumor model. When combined with CpG ODN and ISCOM, recombinant human HCA587 protein induced strong antigen-specific immune responses, which was capable of protecting immunized mice against the challenge of HCA587-expressing B16 melanoma, inhibiting the growth of established tumor and enhancing the survival of tumor-bearing mice.

Selection of appropriate adjuvants is an important consideration in vaccine design. In the case of cancer vaccines, the induction of Th1 type responses is highly desired [34], [35]. In the current study, we found that immunization with HCA587 protein in the absence of adjuvant failed to induce detectable antibody and T cell responses, an observation documented by others in similar studies with the MAGE-3 protein [36], [37]. To improve the efficacy of vaccination, we tested a range of adjuvants, including ISCOM and CpG ODN. ISCOM is a cage-like structure assembled from cholesterol, phospholipids, and saponins. It is featured with the capacity to selectively target antigens to phagocytic cells and enhance the induction of high titer long-lasting antibodies and strong cell-mediated immune responses [30]. CpG ODN, as a ligand of TLR9, induces the activation and maturation of dentritic cells and acts as an adjuvant that switch on Th1 immunity [31], [38]. Both of them have been intensively tested in a number of animal studies and clinical trials, and each shows potent activities to induce integrated humoral and cellular responses to the target antigens. Combination of CpG ODN and ISCOM drastically enhanced the immune response to HCA587 protein, which was strongly biased to the Th1 direction as suggested by the high frequency of IFN-γ-, TNF-α-, and IL-2-producing CD4^+^ T cells and the elevated levels of IFN-γ in serum samples, while each of them alone showed minimal effects.

The increased presence of tumor-infiltrating lymphocytes has been found to correlate with improved clinical outcome in several human cancers [39], [40], [41]. Similarly, we demonstrated that the antitumor immunity conferred by the HCA587 vaccine was associated with drastic changes in the number and composition of the lymphoid population within the tumor. On one hand, there was a marked increase in the total number of intratumoral CD4^+^ T cells and a significant proportion of them were able to produce IFN-γ, TNF-α, and IL-2 upon re-stimulation with the immunizing antigen. On the other hand, we saw a decrease in the percentage of intratumoral Foxp3^+^CD4^+^ Treg cells in the vaccinated mice. As reported by numerous studies, elevated levels of Treg cells in cancer patients usually predicted poor survival and depletion of Treg cells appeared to improve tumor eradication in mouse model [42], [43], [44], [45]. As such, the antitumor effect of the HCA587 protein vaccine may be partly attributable to the alleviation of the immunosuppressive environment of the tumor. Although Th17 cells are also speculated to play a role in antitumor immunity [46], we failed to detect any HCA587-specific, IL-17-producing CD4^+^ T cells in vaccinated mice.

Traditionally, the cytotoxic CD8^+^ T cell is considered to be a major player in immune surveillance, and often serves as the prime target to be activated in cancer immunotherapy [47], [48], [49]. Intriguingly, depletion of CD8^+^ T cells showed no impact on the antitumor immunity elicited by the HCA587 vaccine, which was in contrast to the complete loss of antitumor activity after removal of CD4^+^ T cells. Several recent studies indicated that, in addition to acting as a helper for CD8^+^ T cells, CD4^+^ T cells are able to mediate direct killing of tumor cells in an MHC class II–dependent manner [50], [51]. The exact role of CD4^+^ versus CD8^+^ T cells in antitumor immunity is likely to be dependent on the vaccination strategy and the tumor model to be engaged.

On the molecular level, IFN-γ was found to be a critical mediator for the antitumor effect. After treatment with the HCA587 vaccine, some mice became tumor-free. These mice contained higher numbers of IFN-γ-producing cells than the tumor-bearing mice. As for the tumor-bearing mice, there was clearly a correlation between the number of such cells and the size of the tumor. Further evidence came from studies with IFN-γ-deficient mice, in which the antitumor effect of HCA587 protein vaccine was shown to be completely abrogated. Nevertheless, the mechanism behind IFN-γ-mediated antitumor activity remains to be defined. The current speculation is that IFN-γ exerts the effect possibly through enhancement of cytotoxicity of T cells [51], [52]. Of note, the MHC class II antigen, which is known to be subject to regulation by IFN-γ, was significantly upregulated in the tumor following HCA587 protein vaccine treatment (data not shown), which may render the tumor more vulnerable to CD4^+^ T cells attack.

In summary, the present study demonstrates that the HCA587 protein in combination with ISCOM and CpG OND induces potent antitumor immunity in the mouse. CD4^+^ T cells and IFN-γ are absolutely required for such an activity. This data suggest a potential clinical application of this novel HCA587-based vaccine in the treatment of HCA587-expressing cancer patients.

## Supporting Information

Figure S1 Durability of HCA587-specific cellular and humoral immune responses induced by HCA587 protein vaccine. C57BL/6 mice (3–6 per group) were immunized with HCA587 protein vaccine twice at a 3-week interval or left untreated. Splenoctyes and sera were collected at different points as indicated after the second injection. (A) Number of IFN-γ-producing splenocytes. Splenocytes were restimulated with HCA587 protein for 20 h. IFN-γ-producing cells were detected by ELISPOT assay. Data are presented as mean ± SD. The irrelevant protein ovalbumin (OVA) and medium alone served as controls. (B) Serum antibodies against HCA587 protein. Levels of HCA587-specific antibodies were measured by ELISA. **, *P*<0.01; *, *P*<0.05.
(DOC)

Figure S2 Cytokine production in splenocytes from HCA587 protein vaccine immunized mice. Splenocytes (5×10^6^/ml) from mice (n = 8) vaccinated with HCA587 protein vaccine were cultured in the presence of HCA587 protein (10µg/ml) or OVA (10µg/ml) for 24 h. The supernatants were harvested and assayed by ELISA for IL-4. Data are presented as mean ± SD. ns, *P* > 0.05.
(DOC)

Figure S3 Intracellular cytokine staining of CD4^+^ T cells. Mice were immunized with HCA587+CpG+ISCOM,CpG+ISCOM, or PBS, and boosted 21 days later. Splenocytes were harvested 14 days after the boost and stimulated with HCA587 protein for 24 h. Brefeldin A (10 µg/ml) was added 18 hours before harvesting the cells from the culture. Intracellular staining was performed for IFN-γ and IL-10 by gating on CD4^+^ T cells.
(DOC)

Figure S4 Detection of IgG subclass of anti-HCA587 antibodies in the serum of HCA587 protein vaccine immunized mice. C57BL/6 mice (n = 9) were immunized with HCA587 protein vaccine twice at a 3-week interval. Sera were harvested 14 days after the boost. Levels of HCA587-specific IgG1 and IgG2c were measured by ELISA. Sera were diluted 1∶640,000 before use. **, *P*<0.01.
(DOC)

Figure S5 Detection of HCA587 protein expression in transfected B16 melanoma cells by Western blot. B16 melanoma cells were transfected with plasmid pEGFP-C1-HCA587 or control plasmid pEGFP-C1 (Mock). After selection with G418 and limiting dilution, individual clones were screened for HCA587 expression. The expression of HCA587 protein in a representative clone (D12) was detected by Western blot using anti-HCA587 monoclonal antibody (clone LX-CT10.5).
(DOC)

Figure S6 The specificity of the antitumor effect of HCA587 protein vaccine. C57BL/6 mice (10 per group) were inoculated with 1×10^4^ GFP-expressing B16 (B16-GFP) tumor cells on day 0, and the HCA587 protein vaccine was administrated on day 7 and 28. Tumor size and mouse survival were closely monitored. The tumor volume is presented as mean ± SD on the *left*, and the survival curve is shown on the *right*.
(DOC)

Figure S7 Immunohistochemical staining of mouse testis with anti-HCA587 antibodies. The expression of murine homologous protein of HCA587 was detected by IHC staining using anti-HCA587 antibodies. (A) Negative control with preimmune serum. (B) Positive staining with anti-HCA587 antibodies. Arrow indicates the positive staining cell.
(DOC)
