# Supplementary figures and images for: Induction of HCA587-Specific Antitumor Immunity with HCA587 Protein Formulated with CpG and ISCOM in Mice
Source: PLoS One. 2012 Oct 11;7(10):e47219. doi: 10.1371/journal.pone.0047219 (PMC3469506; doi:10.1371/journal.pone.0047219)

**Figure S1**

**
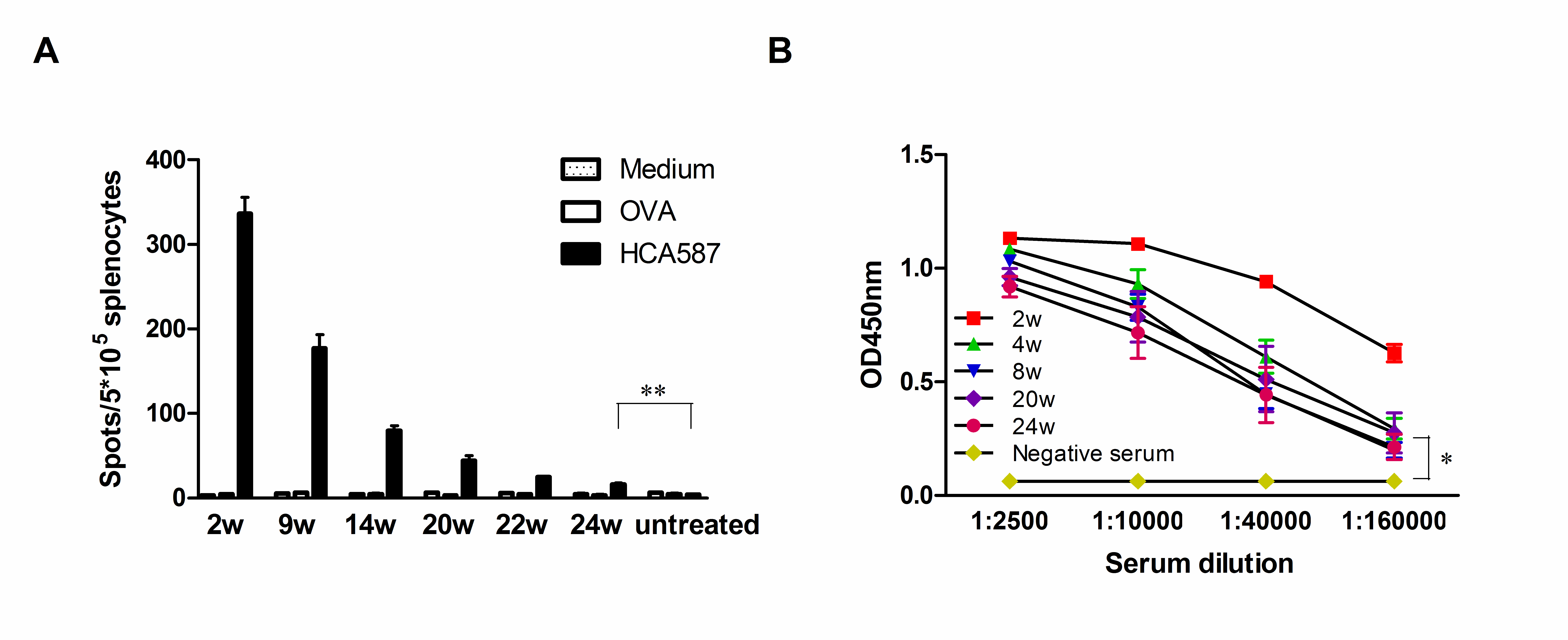
**

Supplement: Figure S1 — Durability of HCA587-specific cellular and humoral immune responses induced by HCA587 protein vaccine. C57BL/6 mice (3–6 per group) were immunized with HCA587 protein vaccine twice at a 3-week interval or left untreated. Splenoctyes and sera were collected at different points as indicated after the second injection. (A) Number of IFN-γ-producing splenocytes. Splenocytes were restimulated with HCA587 protein for 20 h. IFN-γ-producing cells were detected by ELISPOT assay. Data are presented as mean ± SD. The irrelevant protein ovalbumin (OVA) and medium alone served as controls. (B) Serum antibodies against HCA587 protein. Levels of HCA587-specific antibodies were measured by ELISA. **, P<0.01; *, P<0.05. (DOC) [file pone.0047219.s001.doc]

**Figure S2**

**
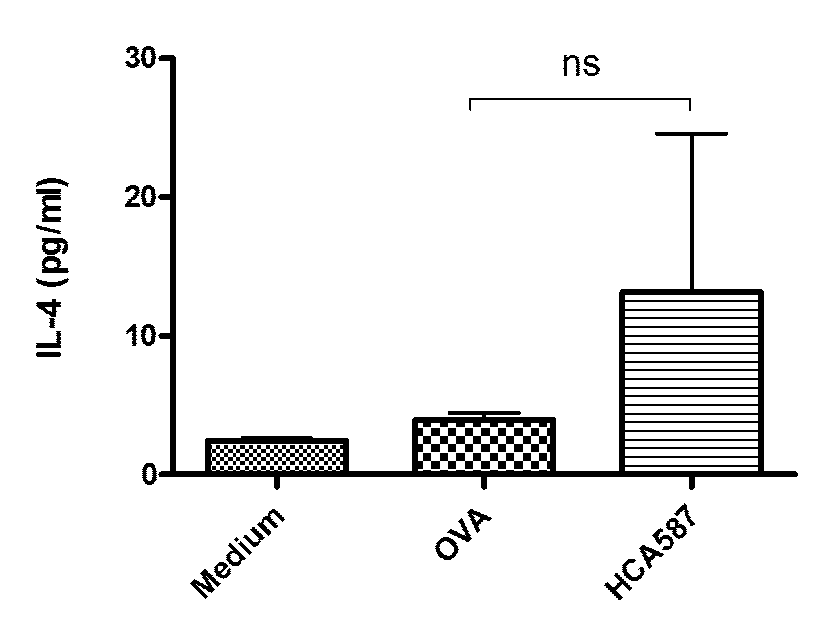
**

Supplement: Figure S2 — Cytokine production in splenocytes from HCA587 protein vaccine immunized mice. Splenocytes (5×10^6^/ml) from mice (n = 8) vaccinated with HCA587 protein vaccine were cultured in the presence of HCA587 protein (10µg/ml) or OVA (10µg/ml) for 24 h. The supernatants were harvested and assayed by ELISA for IL-4. Data are presented as mean ± SD. ns, P > 0.05. (DOC) [file pone.0047219.s002.doc]

**Figure S3**


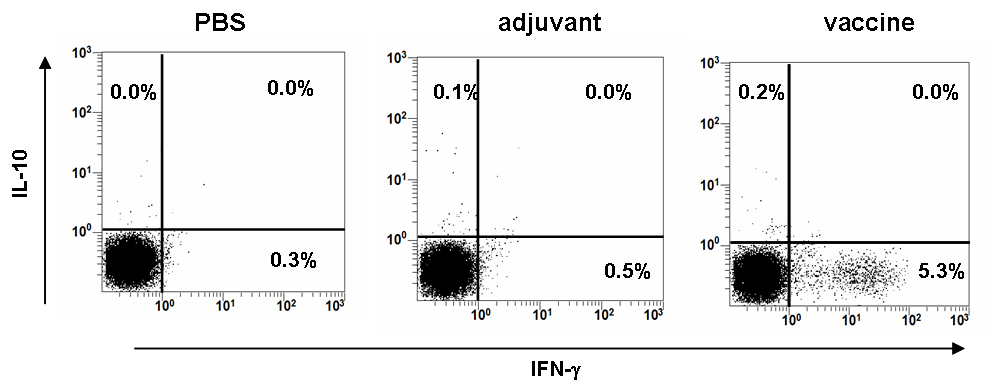

Supplement: Figure S3 — Intracellular cytokine staining of CD4+ T cells. Mice were immunized with HCA587+CpG+ISCOM,CpG+ISCOM, or PBS, and boosted 21 days later. Splenocytes were harvested 14 days after the boost and stimulated with HCA587 protein for 24 h. Brefeldin A (10 µg/ml) was added 18 hours before harvesting the cells from the culture. Intracellular staining was performed for IFN-γ and IL-10 by gating on CD4+ T cells. (DOC) [file pone.0047219.s003.doc]

**Figure S4**

**
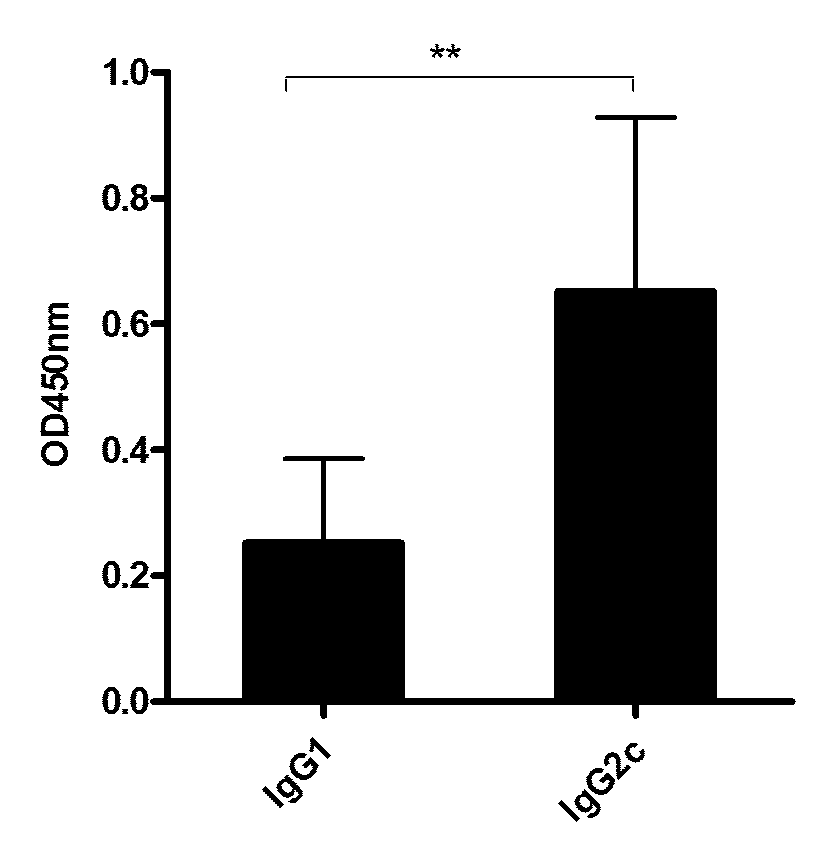
**

Supplement: Figure S4 — Detection of IgG subclass of anti-HCA587 antibodies in the serum of HCA587 protein vaccine immunized mice. C57BL/6 mice (n = 9) were immunized with HCA587 protein vaccine twice at a 3-week interval. Sera were harvested 14 days after the boost. Levels of HCA587-specific IgG1 and IgG2c were measured by ELISA. Sera were diluted 1∶640,000 before use. **, P<0.01. (DOC) [file pone.0047219.s004.doc]

**Figure S5**


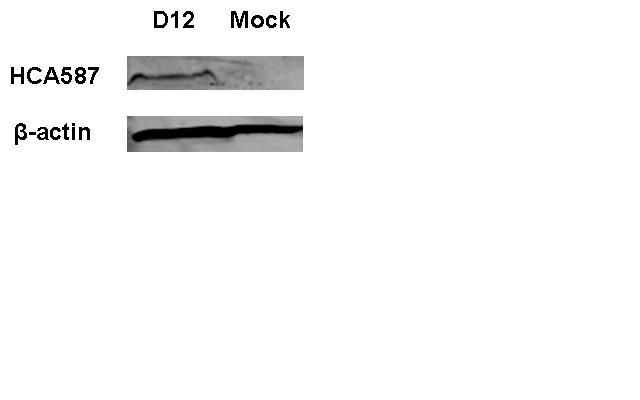

Supplement: Figure S5 — Detection of HCA587 protein expression in transfected B16 melanoma cells by Western blot. B16 melanoma cells were transfected with plasmid pEGFP-C1-HCA587 or control plasmid pEGFP-C1 (Mock). After selection with G418 and limiting dilution, individual clones were screened for HCA587 expression. The expression of HCA587 protein in a representative clone (D12) was detected by Western blot using anti-HCA587 monoclonal antibody (clone LX-CT10.5). (DOC) [file pone.0047219.s005.doc]

**Figure S6**

**
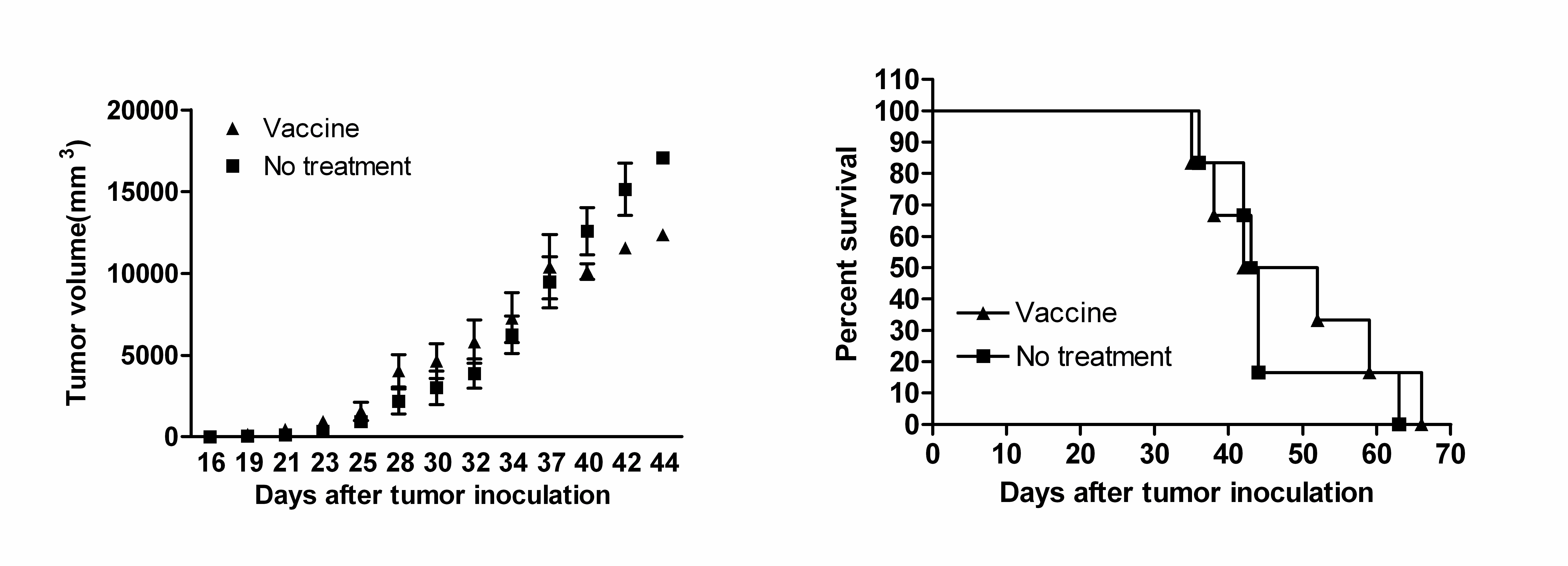
**

Supplement: Figure S6 — The specificity of the antitumor effect of HCA587 protein vaccine. C57BL/6 mice (10 per group) were inoculated with 1×10^4^ GFP-expressing B16 (B16-GFP) tumor cells on day 0, and the HCA587 protein vaccine was administrated on day 7 and 28. Tumor size and mouse survival were closely monitored. The tumor volume is presented as mean ± SD on the left, and the survival curve is shown on the right. (DOC) [file pone.0047219.s006.doc]

**Figure S7**

**A B**


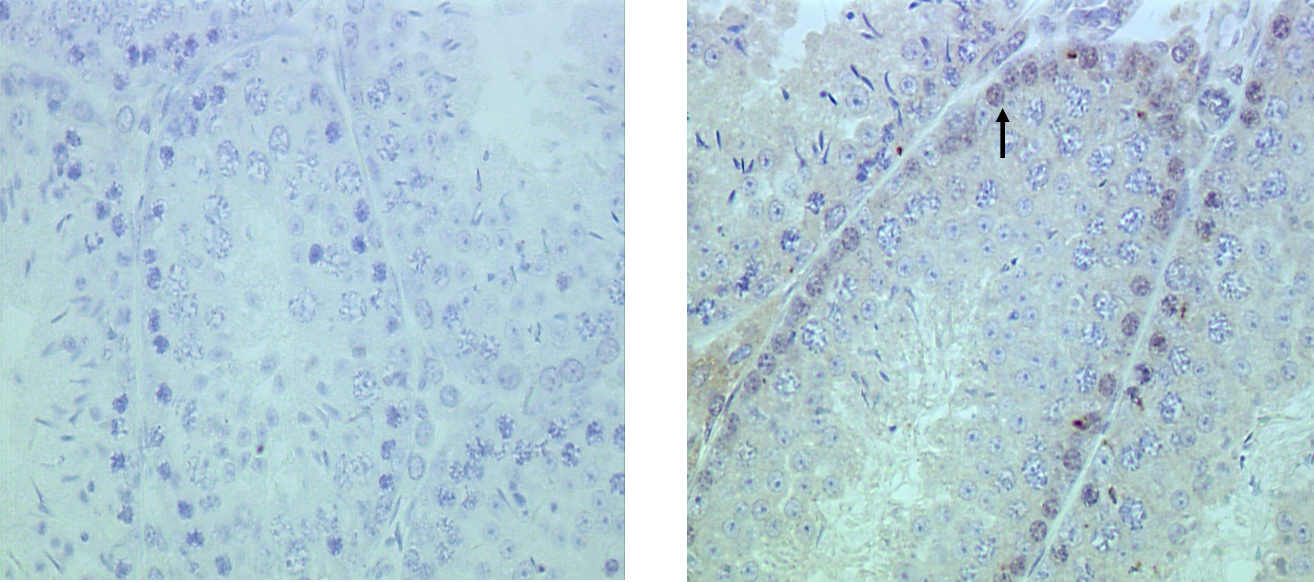

Supplement: Figure S7 — Immunohistochemical staining of mouse testis with anti-HCA587 antibodies. The expression of murine homologous protein of HCA587 was detected by IHC staining using anti-HCA587 antibodies. (A) Negative control with preimmune serum. (B) Positive staining with anti-HCA587 antibodies. Arrow indicates the positive staining cell. (DOC) [file pone.0047219.s007.doc]
